# Matrix enrichment by black phosphorus improves ionization and reproducibility of mass spectrometry of intact cells, peptides, and amino acids

**DOI:** 10.1038/s41598-022-05197-9

**Published:** 2022-01-21

**Authors:** Govinda Mandal, Lukáš Moráň, Lukáš Pečinka, Petr Vaňhara, Josef Havel

**Affiliations:** 1 Department of Chemistry, Faculty of Science, Masaryk University, Kamenice 753/5, 625 00 Brno, Czech Republic; 2 Department of Histology and Embryology, Faculty of Science, Masaryk University, Kamenice 3, 625 00 Brno, Czech Republic; 3 Research Centre for Applied Molecular Oncology, Masaryk Memorial Cancer Institute, Zluty kopec 7, 656 53 Brno, Czech Republic; 4 International Clinical Research Center, St. Anne’s University Hospital, Pekařská 53, 656 91 Brno, Czech Republic

**Keywords:** Stem cells, Chemistry

## Abstract

Intact (whole) cell matrix-assisted laser desorption/ionization mass spectrometry (MALDI-TOF MS) is an established method for biotyping in clinical microbiology as well as for revealing phenotypic shifts in cultured eukaryotic cells. Intact cell MALDI-TOF MS has recently been introduced as a quality control tool for long-term cultures of pluripotent stem cells. Despite the potential this method holds for revealing minute changes in cells, there is still a need for improving the ionization efficiency or peak reproducibility. Here we report for the first time that supplementation by fine particles of black phosphorus to the standard MALDI matrices, such as sinapinic and α-cyano-4-hydroxycinnamic acids enhance intensities of mass spectra of particular amino acids and peptides, presumably by interactions with aromatic groups within the molecules. In addition, the particles of black phosphorus induce the formation of small and regularly dispersed crystals of sinapinic acid and α-cyano-4-hydroxycinnamic acid with the analyte on a steel MALDI target plate. Patterns of mass spectra recorded from intact cells using black phosphorus-enriched matrix were more reproducible and contained peaks of higher intensities when compared to matrix without black phosphorus supplementation. In summary, enrichment of common organic matrices by black phosphorus can improve discrimination data analysis by enhancing peak intensity and reproducibility of mass spectra acquired from intact cells.

## Introduction

Mass spectrometry (MS) of complex biological samples, such as unfractionated cell extracts, intact cells, or body fluids is challenging due to the inherent structural and chemical complexity of biomolecules within cells, and their mutual interactions. In addition, fragmentation or poor ionization of analytes can limit the detection threshold and compromise the reproducibility of results. The introduction of matrix-assisted laser desorption/ionization time-of-flight (MALDI-TOF) revolutionized bioanalytical chemistry and a panel of mostly organic matrices is commonly used for soft ionization of analytes. Enrichment of matrix by inorganic elements, such as gold or phosphorus^1^ can improve the ionization efficacy and in the case of monoisotopic elements, also can provide a suitable tool for internal calibration^2^. However, the effects of such inorganic additives to standard MALDI matrix have not been sufficiently addressed.

Elemental phosphorus forms various allotropes that differ in their structure. The black phosphorus (BP) is a highly stable allotrope that can form homoatomic supermolecular structures, e.g., cages or layers. It has been described by He et al. recently^3^, that BP enhances ionization of analytes labelled by quaternary ammonium groups, most probably by nanoparticle- or surface-associated effects. In addition, this approach was claimed to be highly suitable for analysis of body fluids, such as saliva, urine, or blood serum, and quantitation of aldehyde species therein. Similarly, Wang et al. proposed a rapid and sensitive method for the determination of chemical labelled glucose serum using black phosphorus as a matrix in time-of-flight mass spectrometry^4^. It is highly probable, that phosphorus effects in MS are linked to its nanostructure. Other allotropes were also reported to be relevant in mass spectrometry applications, including red phosphorus and phosphorene. An insight on stability and homoatomic interactions of elemental phosphorus in MS was previously provided by Sládková et al.^5^ who investigated the generation of phosphorus clusters P_*n*_ (*n* = 1–89) from red phosphorus covering the range of mass up to 3000 m*/z* by applying laser desorption ionization (LDI) technique in both positive and negative ion modes and proposed that clusters of red phosphorus can serve as a useful calibration tool in MS.

In addition, red phosphorus provided a suitable matrix for the detection of peptides and proteins^5^. Phosphorene can be prepared by liquid exfoliation from black phosphorus. It forms thin, two-dimensional layers, similar to graphene, but with distinct properties^6,7^. Rubio-Pereda et al*.*^8^ investigated how phosphorene and amino acids interact by density functional theory calculation. It was suggested that this interaction is mediated by the electron transfer process from phosphorene to amino and carboxylic functional groups of amino acid molecules.

The adsorption–desorption behaviour of black phosphorus quantum dots to the mucin surface strongly depends on the pH value, ionic strength, and ionic valence^9^. The functionalization of black phosphorus quantum dots with one or more benzene rings or anthracene was studied and suggested that a red shift of absorption is found in the absorption spectra. This red shift is a result of electron delocalization in the BP/organic molecule nanostructure^10^. The black phosphorus quantum dots can also be functionalized noncovalently with organic moieties^11^. Also, BP nanoparticles interact with proteins (bovine serum albumin and bovine haemoglobin) resulting in alteration of their tertiary structure^12^.

Here we report that enrichment of sinapinic acid (SA) and α-cyano-4-hydroxycinnamic acid (CHCA) matrix by fine particles of black phosphorus improves crystallization of matrix, ionization, and reproducibility of MALDI-TOF MS of intact cells, proteins, peptides, and amino acids.

## Results and discussion

### BP-enriched SA MALDI matrix improves intact cell MALDI-TOF MS

We have recently demonstrated that intact cell MALDI-TOF MS can provide a useful tool for the quality control in long-term cultures of human embryonic stem cells (hESCs). Alterations in cells induced by culture conditions can prevent safe biomedical or clinical applications of hESCs, due to the development of unwanted or even hazardous phenotypic traits^13^. These cellular alterations can be correlated with specific changes in mass spectra^17^. However, recognition of spectral patterns and discrimination of cell types or states based on specific spectral signatures depends on the matrix composition and ionization of analytes that can affect data analysis. Here we examined the effects of BP on intact cell mass spectrometry using SA matrix. To model subtle differences in cells, we used hESC line CCTL14 of low passage number (P35), representing non-aberrant cells, and culture-adapted, high passage number (P307) cells. As a distant biological control, the human ovarian cancer cell line SKOV-3 was used.

**Figure 1:**
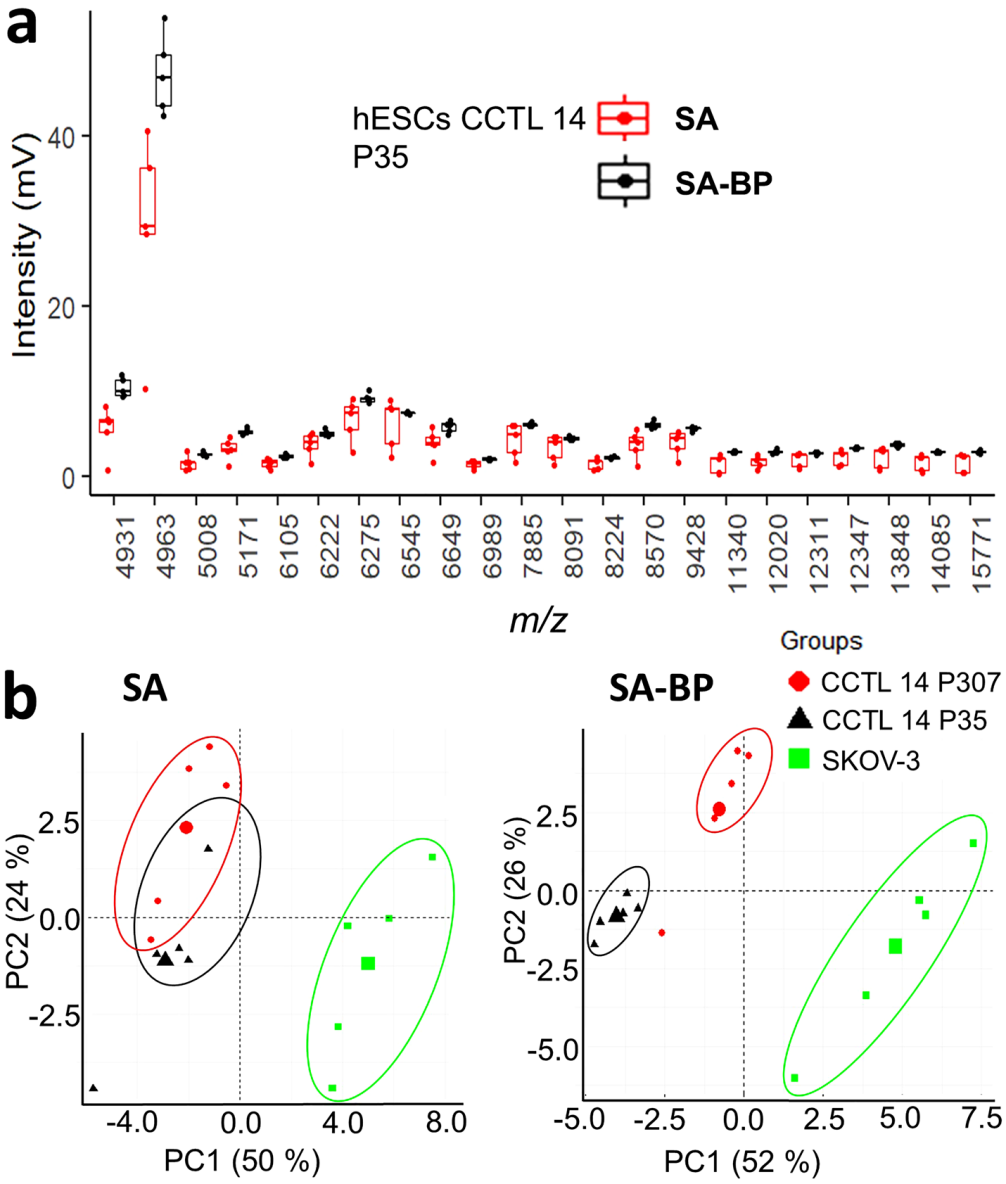
Enrichment of sinapinic matrix acid by black phosphorus improves reproducibility of mass spectra and discrimination analysis. (**a**) Averaged peak intensities (*n* = 5) acquired from intact human embryonic stem cell line CCTL 14, passage number (P) 35. Box-plots show medians ± 25 and 75 quartiles of peak intensities obtained either with sinapinic acid (SA) matrix only (red) and with SA matrix enriched by black phosphorus (BP) (black). Outliers are defined as 1.5× interquartile range, (**b**) Principal component analysis (PCA) of spectral profiles acquired from hESCs CCTL 14 of P35 and P307, and ovarian cancer cell line SKOV3. Intact cell MALDI TOF MS was performed either using the SA matrix alone or SA matrix enriched by BP, in positive ion mode with laser energy 140 a.u. 100 spectral profiles were recorded.

Laser power has been optimized for the optimal ionization of biomolecules from intact cells using SA and SA-BP matrices (Fig. S1). Laser power ranging 130–160 a.u. provided optimal signals. Laser power 140 a.u. was then used for intact cell MALDI-TOF MS. Interestingly, the intensities of mass spectra were higher in BP-enriched matrix when compared to with SA only. Mass spectra were recorded in five technical replicates for each biological sample, normalized, and used for descriptive statistics and multivariate data analysis. Only peaks (*m*/*z* value) with signal to noise ratio over five (S/N > 5) were used for analysis. These peaks represented ~ 20% of all peaks in average mass spectrum. The addition of the BP to SA matrix significantly and selectively increased the intensity of most of the peaks, and also decreased the variability of peak intensities between replicates (Fig. 1a). The representative mass spectra recorded using SA and SA-BP matrices are shown in Fig. S2. The spectral data were then used as inputs for principal component analysis (PCA). As a proof of principle, we used hESC line CCTL 14 of low and high passage numbers corresponding to the shifts of phenotype in long-term in vitro cultures.

Mass spectra recorded from intact cells using BP-enriched matrix, provided better inputs for principal component analysis (Fig. 1b) that clustered clearly even biologically close samples, such as hESCs cultured for different time (number of passages). Taken together, we demonstrated that enrichment of SA matrix by fine particles of BP improved ionization of molecules from intact cells and provided more reproducible spectral patterns.

### BP-enriched SA matrix produces smaller and homogenous crystals

**Figure 2:**
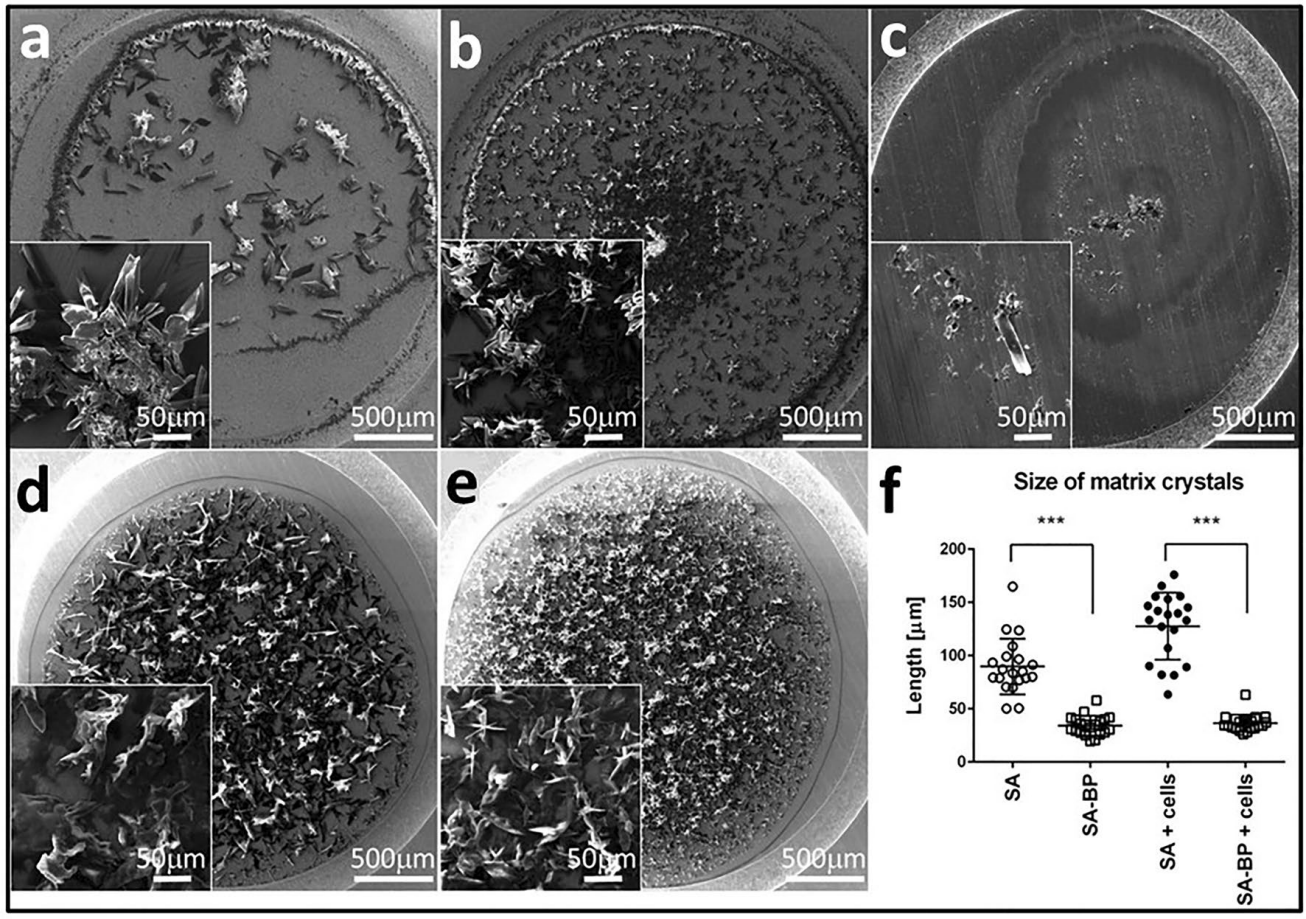
Black phosphorus improves sinapinic matrix crystallization as visualized by scanning electron microscopy (SEM). SA matrix either alone or enriched by BP was mixed with cells washed in 1× phosphate-buffered saline and resuspended in 150 mM ammonium bicarbonate buffer, spotted in the steel MALDI plate, and visualized by SEM. (**a**) SA only, (**b**) SA enriched by BP (SA-BP), (**c**) BP suspension only, (**d**) SA + hESC cell line CCTL L14, (**e**) SA-BP + hESC cell line CCTL L14, and (**f**) size distribution of matrix crystals (*n* = 20).

Crystallization of the MALDI matrix is a complex process that has a significant effect on desorption and ionization, and therefore on the quality of results^14^. Various additives have been shown to improve MALDI-TOF MS of lipids, proteins, and other biomolecules^15,16^. Here we were curious if the addition of fine particles of BP affects the formation of SA crystals. We have deposited the BP-enriched SA matrix onto the steel MALDI target plate and visualized the crystals by scanning electron microscopy (SEM). Crystals of SA enriched by BP were significantly smaller and homogenously dispersed over the target plate spot when compared to SA. When we mixed SA enriched by BP with non-lysed cells according to the protocol for intact cell MALDI-TOF MS^17^ the SEM revealed that the BP-enriched SA matrix formed uniform, small-sized crystals, regularly covering the deposited cells (Fig. 2).

### BP enhances ionization of amino acids and peptides

According to the literature, the CHCA matrix was used for MALDI-TOF MS of selected amino acids and peptides^1,18^. Then, we optimized the concentration of amino acids and peptides and their deposition on the target plate for optimum ionization (Fig. S3). After optimization, a 1 µL mixture of analyte and matrix (CHCA or SA) either with or without BP deposited on target and then dried before MS analyses.

**Figure 3:**
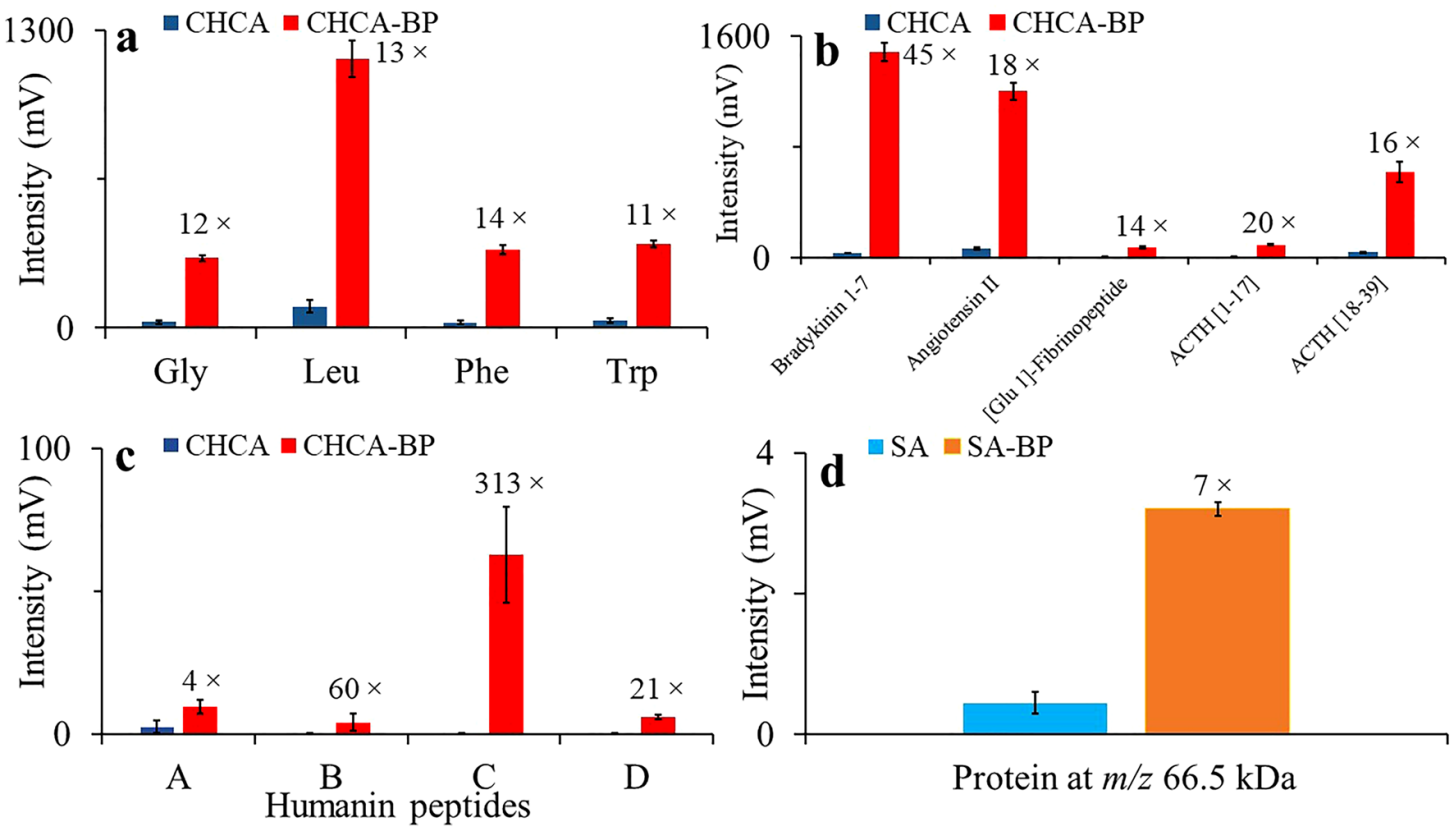
Black phosphorus improves peak intensity of amino acids, peptides, and proteins. (**a**) amino acids (intensity increment: 11–14 times, *n* = 4), (**b**) standard peptides (intensity increment: 14–45 times, *n* = 4), (**c**) humanin peptides (intensity increment: 4–313 times, *n* = 4), and (**d**) bovine serum albumin (BSA, intensity increment: 7 times, *n* = 5) using CHCA or SA matrix either alone or enriched by black phosphorus. Mass spectra were recorded in positive ion mode, laser energy 110 a.u. for amino acids and peptides and 180 a.u. for BSA, respectively. 100 spectral profiles were recorded.

**Figure 4:**
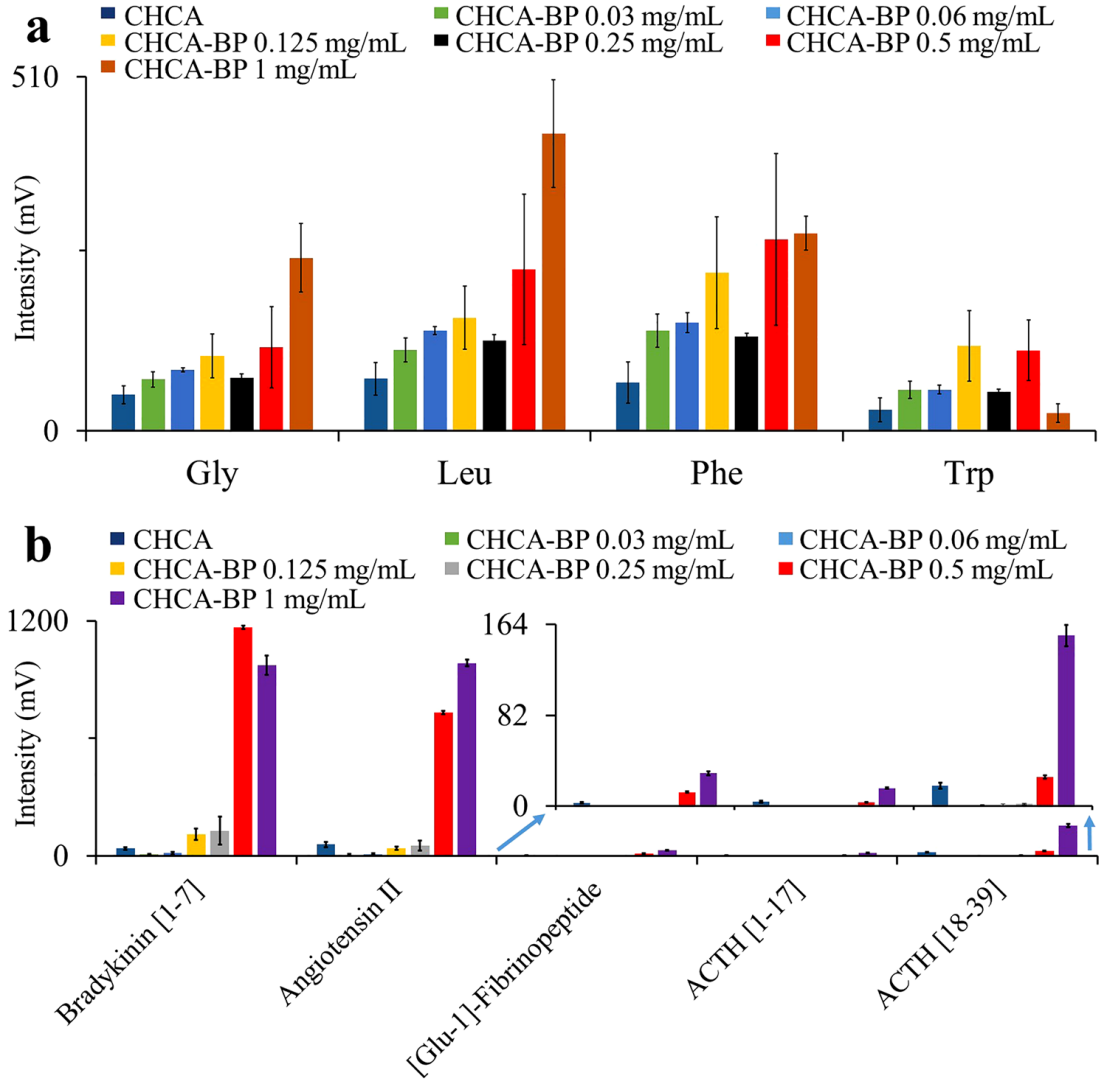
Effect of BP on peak intensity is dose dependent. Matrix solution containing α-cyano-4-hydroxycinnamic acid (CHCA) was supplement with increasing concentration of BP as indicated. (**a**) amino acids, (**b**) peptides. Mass spectra were recorded in positive ion mode with laser energy 70 a.u. for amino acids and 110 a.u. for peptides, respectively. 100 spectral profiles were recorded. Columns show means ± Std. errors of three independent experiments.

Intensities of mass spectra generated from glycine, leucine, phenylalanine, and tryptophan amino acids were significantly increased (Fig. 3a). The effect of matrix enrichment was even more prominent in various peptides including, bradykinin [1–7], angiotensin II, [glu-1]-fibrinopeptide, ACTH [1–17], ACTH [18–39], and humanin peptides A, B, C, and D when BP-enriched CHCA matrix was used. Intensities of mass spectra recorded from standard peptides and humanin peptides A, B, C, and D are shown in Fig. 3b,c. Interestingly, the increased peak intensity was observed also in the case of bovine serum albumin, a 66.5 kDa protein (Fig. 3d). The representative mass spectra recorded from amino acids, peptides, and BSA using matrix only and BP-enriched matrix are shown in Fig. S4. In summary, the BP-enriched CHCA matrix yielded higher intensity of amino acids and peptides peaks than CHCA matrix only. The effect of BP was clearly dose-dependent (Fig. 4). In addition, the representative mass spectra recorded from amino acids and peptides using CHCA and CHCA-BP matrices are given in Fig. S5.

### BP interacts with amino acids and peptides

**Figure 5:**
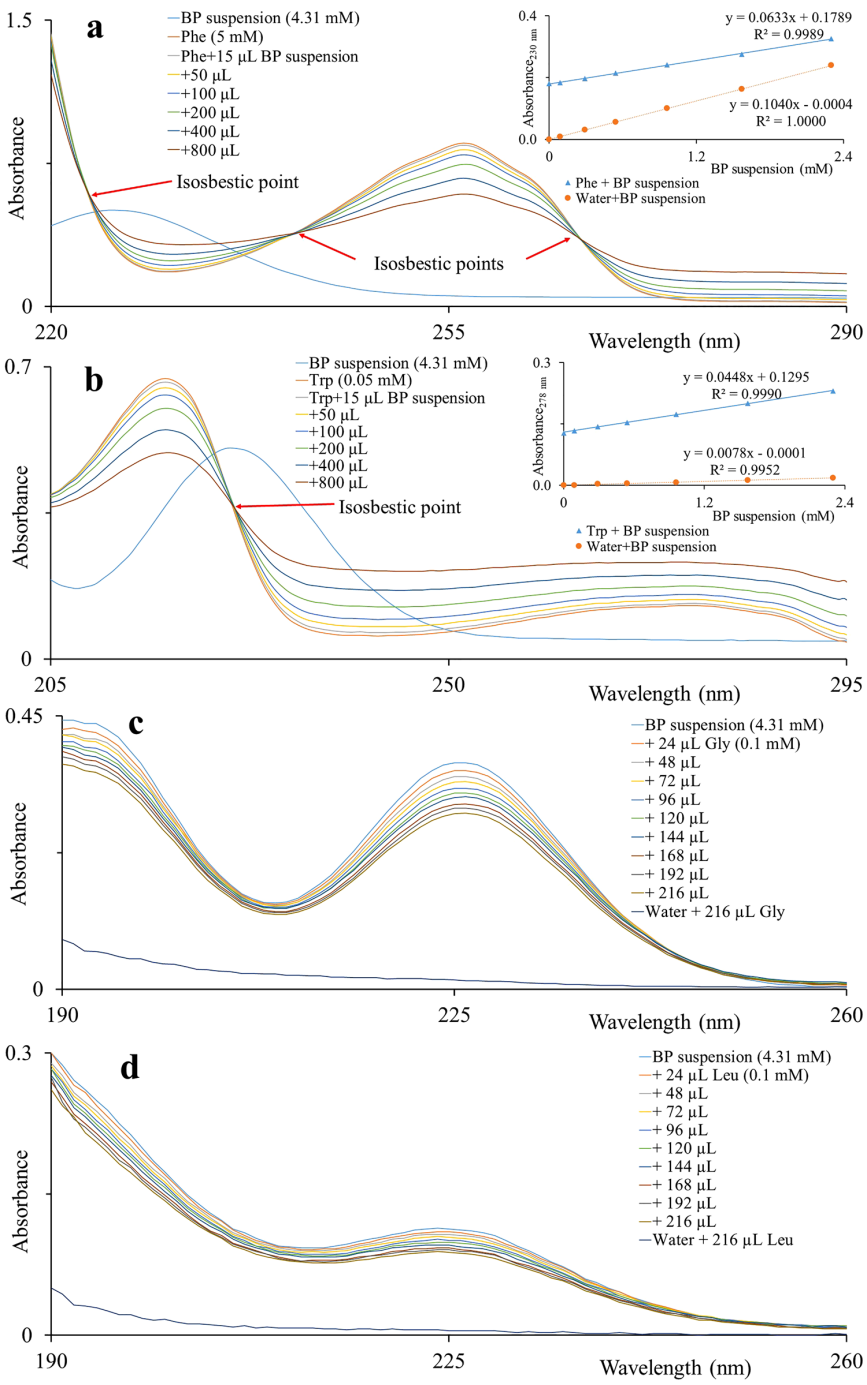
Black phosphorus preferentially interacts with amino acids containing aromatic group. Amino acids water solutions were titrated with BP water suspension and absorption spectra were recorded. (**a**) phenylalanine, (**b**) tryptophan, (**c**) glycine, and (**d**) leucine. The inset shows the absorbance at 230 and 278 nm wavelengths plotted against the concentration of BP fine particles in the respective solution (blue) and in water (orange).

To elucidate that BP indeed physically interacts with the amino acids and peptides, we performed the UV–VIS spectroscopy and fluorimetry analysis. We hypothesised that the presence of aromatic rings in amino acid structure can be responsible for the observed effects. Therefore, we first measured the absorbance of aromatic amino acids (Phe, Trp) and then titrated them with the suspension of fine BP particles. Four absorption bands were observed with three isosbestic points in the spectra of Phe (Fig. 5a). Two absorption bands were observed with an isosbestic point in spectra of Trp (Fig. 5b). In the case of Gly and Leu, there was no increase of absorbance or no isosbestic points in absorption spectra after titration (Fig. 5c,d).

**Figure 6:**
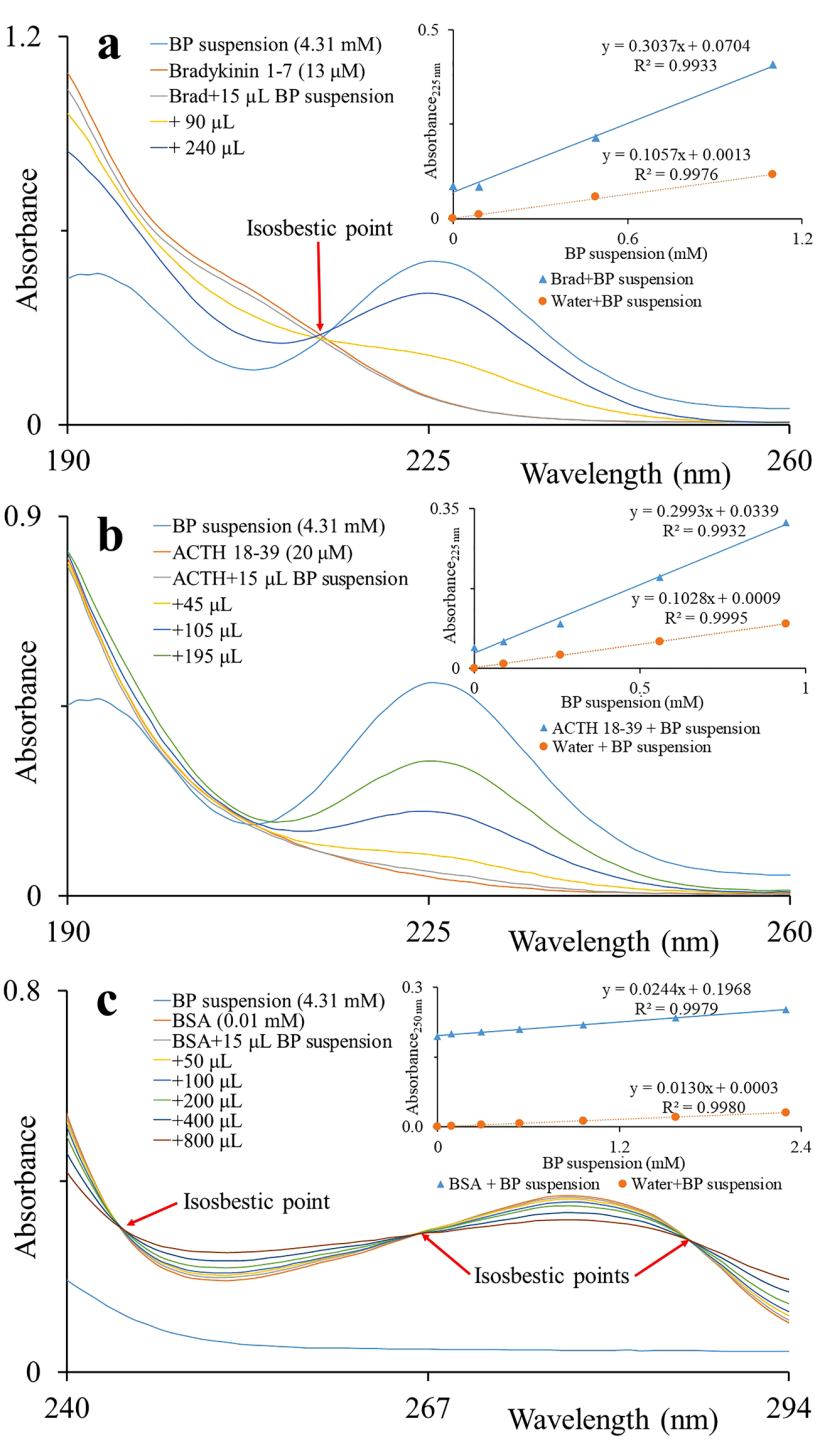
Black phosphorus interacts with peptides. Water solutions of bradykinin fragment [1–7], adrenocorticotrophic hormone fragment [18–39] and bovine serum albumin were titrated with BP water suspension. Absorption spectra were recorded. (**a**) bradykinin [1–7], (**b**) ACTH [18–39], and (**c**) BSA solutions. The inset shows the absorbance at 225 and 250 nm wavelengths plotted against the concentration of BP fine particles in the respective analyte solution (blue) and in water (orange).

Similar effects were observed for solutions of bradykinin [1–7] and ACTH [18–39] with BP addition. (Fig. 6a,b). An isosbestic point and absorption maximum at 225 nm were observed in spectra of bradykinin [1–7]. Therefore, it indicates the interaction between bradykinin and BP fine particles. On the other hand, only absorption maximum at 225 nm was observed rather than an isosbestic point in spectra of ACTH [18–39]. The solution of BSA was titrated with BP suspension and absorption spectra of resulted mixtures were measured (Fig. 6c). Four absorption bands and three isosbestic points were observed in the spectra of BSA.

We have also investigated the interaction of SA matrix with BP fine particles. The solution of SA matrix was titrated with BP suspension and absorbance spectra were measured. However, there were no absorbance bands or no isosbestic points in absorption spectra observed after the incremental addition of BP. Therefore, we suggest that there is no interaction between BP fine particles and SA matrix (data not shown).

Then, we employed fluorimetry to validate the possible interactions of aromatic amino acids with BP particles. The suspension of fine BP particles was gradually titrated increasing the concentration of either Phe or Trp solution and fluorescence/emission spectra were measured. We observed a single peak in the emission spectrum at 356 nm that incrementally increased in a dose-dependent manner (Fig. S6).

To explain the interaction statistically, we have determined the regression lines from the plots of absorbance against the concentration of BP fine particles in respective analyte solutions and in water. In the case of interactions, the linear regression line (and slope) obtained from the analyte solution should not be parallel with that line obtained from water and vice-versa. Due to the interaction, the absorbance is not increased or decreased in the same way (analyte + BP) as in the blank (water + BP). The slopes of regression lines obtained from the plots of absorbance against the concentration of BP particles in analytes solutions are quite different from those obtained from the plots of absorbance against the concentration of BP particles in water. Based on the above facts, it proves that BP fine particles interact with selected biomolecules. Even though, these amino acids (without aromatic ring) are ionized significantly in MS while using BP-enriched CHCA matrix. It indicates that there is interaction not only due to aromatic rings of biomolecules which is responsible for enhanced ionization in MS. So, one can suggest that interactions between different biomolecules and BP particles might be due to van der Waals forces, electron transfer forces, hydrophobic forces, and/or π-π bonding^12,19^.

The mechanism of how the BP particles interact with the amino acids, peptides, proteins, or substances within eukaryotic cells remains elusive. Here, we propose that the observed increase of ionization in MALDI-TOF MS using BP-enriched matrices can involve: (i) BP transfer laser energy to analyte more efficiently than common organic matrix, (ii) BP eliminate the competition of analyte and matrix during ionization process, (iii) formation of smaller crystals with more homogenous distribution of analytes on target, (iv) interactions between BP and analyte are very weak (π-π, van der Waals interactions, etc.), resulting in efficient desorption (SALDI effect), and (v) the delocalization of electrons in BP/biomolecules nanostructure^10^ decreases the density of electrons in plasma plume and hence increases the availability of protons for the formation of sufficient protonated peptides and therefore enhanced ionization.

## Conclusions

Here we demonstrated for the first time that the enrichment of common MALDI matrices (SA, CHCA) by BP improves intact-cells MALDI-TOF MS by enhanced ionization of peptides and amino acids. Mass spectra of intact cells acquired using BP-enriched matrix provided improved inputs for discrimination data analysis. The BP promoted formation of small and regularly dispersed crystals of samples on the steel MALDI plate and reduced inconsistencies in matrix/analyte deposition. We suggest that amino acids (Phe and Trp) containing aromatic rings, either alone or in peptides and proteins are preferentially interacting with surface of BP fine particles, while those without aromatic rings (Gly and Leu) are not. In summary, enrichment of organic matrices by BP improves outputs of intact cell MALDI-TOF MS.

## Materials and methods

### Chemicals

Water was double distilled using quartz apparatus from Heraeus Quarzschmelze (Hanau, Germany). Acetonitrile, glycine (Gly), and leucine (Leu) were bought from PENTA (Praha, Czech Republic). Trifluoroacetic acid (TFA), α-cyano-4-hydroxycinnamic acid (CHCA), ammonium bicarbonate (ABC), 3,5 dimethoxy-4-hydroxycinnamic acid (sinapinic acid, SA), tryptophan (Trp), and bovine serum albumin (BSA) were purchased from Sigma-Aldrich (Steinheim, Germany). Black phosphorus was purchased from Chempur (Karlsruhe, Germany). Phenylalanine (Phe) was purchased from Merck (Darmstadt, Germany). Bradykinin fragment 1–7, angiotensin II, [glu^1^]-fibrinopeptide, adrenocorticotropic hormone [1–17] (ACTH [1–17]), and adrenocorticotropic hormone [18–39] (ACTH [18–39]) were purchased from Sigma-Aldrich (Steinheim, Germany). Humanin peptides A (C_84_H_138_N_20_O_24_S, PRGFSCLLLLTGEIDLP), B (C_83_H_136_N_20_O_23_S_2_, PRGFSCLLLLCGEIDLP), C (C_85_H_140_N_20_O_25_S, PRGFSCLLLLTSEIDLP), and D (C_118_H_202_N_34_O_31_S_2_, MAPRGFSCLLLLTGEIDLPVKRRA) were purchased from Clonestar biotech (Brno, Czech Republic). CCTL14 line of hESC [46, XX] registered in Human Pluripotent Stem Cell Registry was used in this study. This hESC line was kindly provided by Dr. Aleš Hampl, Ph.D. from the Department of Histology and Embryology, Faculty of Medicine, Masaryk University, and used in consonance with the official permission (MSMT-14648/2016-5).

### Instrumentation

Mass spectra were recorded using AXIMA CFR MALDI-TOF Mass Spectrometer from Kratos Analytical Ltd. (Manchester, UK), equipped with a nitrogen laser (337 nm). The laser energy was selected from 0 to 180 a.u*.* (arbitrary units). The repetition mode of experiments was performed at a frequency of 5 Hz and a pulse time width of 3 ns. Analyses were carried out in linear/reflectron positive/negative ion modes. Generally, MALDI-TOF MS of amino acids and peptides is usually measured in the positive ion mode, therefore, the study was performed in the positive ion mode. The irradiated spot size was about 150 µm in diameter and the maximum laser power was 6 mW (180 a.u.). The resulting laser energy fluence was ~ 1 J/cm^2^.

UV–Visible spectra were measured using a UV mini-1240 spectrophotometer from Shimadzu Corporation (Kyoto, Japan). A spectrofluorometer Aminco-Bowman Series 2 (Thermo Fisher Scientific, MA, USA) equipped with a 150 W Xe lamp was used. Excitation wavelength was set at 445 nm and emission wavelength was set at 525 nm. The spectra were acquired with 1 nm resolution and a scanning speed of 1 nm/s. A MiniSpin Plus of Eppendorf (Hamburg, Germany) centrifuge machine was used for centrifugation.

### Preparation of BP suspension for UV–Vis spectrophotometry and fluorimetry

Bulk BP was crushed using mortar and pestle and 5 mg of crushed powder was suspended in 2 mL of water. The suspension was sonicated for 15 min. After sonication, the suspension was centrifuged for 5 min at 14,500 rpm. The supernatant of BP suspension containing BP fine particles was used. The concentration of BP fine particles in the supernatant of BP suspension was determined gravimetrically as 4.31 ± 0.54 mM on average from three determinations (*n* = 3, standard error of mean = 0.54). The average size of BP fine particles in suspension was 300 nm as determined by transmission electron microscopy (data not shown).

### Cell culture

Undifferentiated human embryonic stem cells (hESCs) line CCTL 14 (hPSCreg: MUNIe007-A, RRID;CVCL_C860) were maintained in 6-cm Petri dishes (TPP, Trasadingen, Switzerland) on mitotically inactivated mouse embryonic fibroblasts (MEFs) in Dulbecco’s Modified Eagle’s Medium (DMEM)/F12 supplemented with 15% knockout serum replacement (both from Invitrogen, Life Technologies, Carlsbad, CA), l-glutamine, minimum essential medium, nonessential amino acids, 0.5% penicillin–streptomycin (PAA Laboratories, Pasching, Austria), 2-mercaptoethanol (Sigma-Aldrich, Prague, Czech Republic), and 10 ng/mL fibroblast growth factor-2 (PeproTech, Rocky Hill, NJ). Ovarian cancer cell line SKOV-3 was cultured in DMEM enriched with 10% fetal calf serum (FCS) (Prague, Czech Republic) and 1% penicillin–streptomycin (PAA Laboratories, Pasching, Austria). Cells were maintained in an incubator at 37 °C in a humidified atmosphere containing 5% CO_2_ and the media was exchanged daily (hESCs) or every two or three days (SKOV-3).

### Sample preparation for UV–Vis spectrophotometry and fluorimetry

BSA solution was prepared in water (0.01 mM). Amino acids solutions Gly (0.1 mM), Leu (0.1 mM), Phe (5 mM), and Trp (0.05 mM) were prepared in water. Solution of Bradykinin fragment [1–7] was prepared by diluting 10 µL of bradykinin fragment [1–7] solution (1 mg/mL solution in 0.1% TFA) to 1 mL of water getting 13 µM stock solution. ACTH [18–39] solution was prepared by diluting 50 µL of ACTH [18–39] solution (1 mg/mL solution in 0.1% TFA) to 1 mL of water getting 20 µM stock solution. Finally, solutions of analytes were gradually titrated with supernatant of BP suspension in the final volume of 700 μL. Absorption spectra were then measured. For fluorimetry, Phe (0.01 mM) and Trp (0.01 mM) solutions were prepared in water. Supernatant of BP suspension was gradually titrated with analyte solutions in the total volume of 700 μL. Fluorescence/emission spectra were measured.

### Sample preparation for mass spectrometry

Intact, non-lysed cells were harvested, washed two times with ice-cold phosphate-buffered saline (PBS), resuspended in 150 mM ammonium bicarbonate (ABC) buffer filtered through a 0.22-micron membrane filter. 10 µL of cell suspension were mixed with 5 µL of SA or BP-enriched SA matrix. 25 × 10^3^ of cells per measurement were used. The SA matrix solution for intact cells MS was prepared by dissolving 30 mg SA in 1 mL of solvent mixture (700 µL acetonitrile, 225 µL water, and 75 µL TFA). Stock solution of bovine serum albumin (BSA) was prepared in water (0.05 mM) and mixed with the SA matrix to the final concentration of 0.005 mM (1:10). The SA matrix solution for BSA analysis was prepared by dissolving 20 mg SA in 1 mL of solvent (500 µL acetonitrile, 497 µL water, and 3 µL TFA). Amino acids stock solutions Gly, Leu, Phe, and Trp were prepared in water (100 mM) and mixed with CHCA matrix to the final concentration of 100 µM (1:10). Stock solutions of standard peptides (bradykinin fragment 1–7, angiotensin II, [glu-1]-fibrinopeptide, ACTH 1–17, and ACTH 18–39) were prepared in 0.1% TFA solution in water (1 mg/mL) and mixed with CHCA matrix to the final concentration of 0.2 mg/mL (1:10). Stock solutions of humanin peptides were prepared in 0.1% TFA solution in water (2 mg/mL) and mixed with CHCA matrix to the final concentration of 0.02 mg/mL (1:10). The amino acids and peptides were analysed using CHCA matrix solution prepared by dissolving 10 mg CHCA in 1 mL of solvent mixture (500 µL acetonitrile, 499 µL water, and 1 µL TFA). For all analytes except the intact cells, 1 µL aliquot was deposited on the MALDI steel target plate and allowed to dry. In the case of cells, 2 µL aliquot was deposited on target and allowed for drying. Finally, the MALDI steel target plate was introduced into the mass spectrometer. Mass spectra were recorded when the level of vacuum was below 4 × 10^–5^ Pa.

The BP suspension was prepared by suspending 1 mg BP powder in 1000 µL acetonitrile. The BP-enriched CHCA or SA matrices were prepared by suspending 1 mg BP powder in 1 mL of prepared CHCA or SA matrix solution. All matrices were sonicated for 10 min before use.

Sample deposition on target: five methods were examined, (i) sample-first method (order of sample deposition: 1 µL analyte, 1 µL matrix, drying), (ii) matrix-first method (order of sample deposition: 1 µL matrix, 1 µL analyte, drying), and (iii) three-layer method or sandwich (order of sample deposition: 1 µL matrix, 1 µL analyte, 1 µL matrix, drying), (iv) sandwich (order of sample deposition: 1 µL matrix and drying, 1 µL analyte and drying, 1 µL matrix and drying) as described in literature^20^, and (v) mixture (order of sample deposition: 1 µL of mixture (matrix + analyte), drying). Deposition of the premade mixture of analyte and matrix provided the most consistent results.

### Scanning electron microscopy

SA solution, or SA solution enriched by BP either with or without intact cells, or BP suspension alone, was deposited on MALDI steel target plate and analysed by TESCAN VEGA TS 5136 XM. Image analysis and measurement of crystal parameters were performed in ImageJ software (U. S. National Institutes of Health, Bethesda, Maryland, USA).

### Software and computation

Launchpad software (Kompact, version 2.3.4, 2003) from Kratos Analytical Ltd (Manchester, UK) was used to analyse mass spectra. All statistical computations were done in the R programming environment. Plots were visualized in GraphPad (GraphPad Software Inc., San Diego, CA, USA).

## Supplementary Information

Supplementary Figures.

## Data Availability

The datasets generated and analysed during the current study are available from the corresponding author on reasonable request.
