# Supplementary material for: Matrix enrichment by black phosphorus improves ionization and reproducibility of mass spectrometry of intact cells, peptides, and amino acids
Source: Sci Rep. 2022 Jan 21;12:1175. doi: 10.1038/s41598-022-05197-9 (PMC8782824; doi:10.1038/s41598-022-05197-9)
Supplement: Supplementary file 1 — Supplementary Figures. [file 41598_2022_5197_MOESM1_ESM.pdf]

Supplementary Information

Matrix enrichment by black phosphorus improves ionization and reproducibility of mass spectrometry of intact cells, peptides, and amino acids

Govinda Mandal[1#], Lukáš Moráň[2,3#], Lukáš Pečinka[1,4#], Petr Vaňhara[2,4], Josef Havel[1,4*]

[1]Department of Chemistry, Faculty of Science, Masaryk University, Kamenice 753/5, 625 00 Brno, Czech Republic. [2]Department of Histology and Embryology, Faculty of Science, Masaryk University, Kamenice 3, 625 00 Brno, Czech Republic. Research Centre for Applied Molecular Oncology, Masaryk Memorial Cancer Institute, Zluty kopec 7, 656 53, Brno, Czech Republic, [4]International Clinical Research Center, St. Anne's University Hospital, Pekařská 53, 656 91 Brno, Czech Republic. [#]these authors contributed equally. [*]email: havel@chemi.muni.cz

Correspondence to: Josef Havel; e-mail: havel@chemi.muni.cz

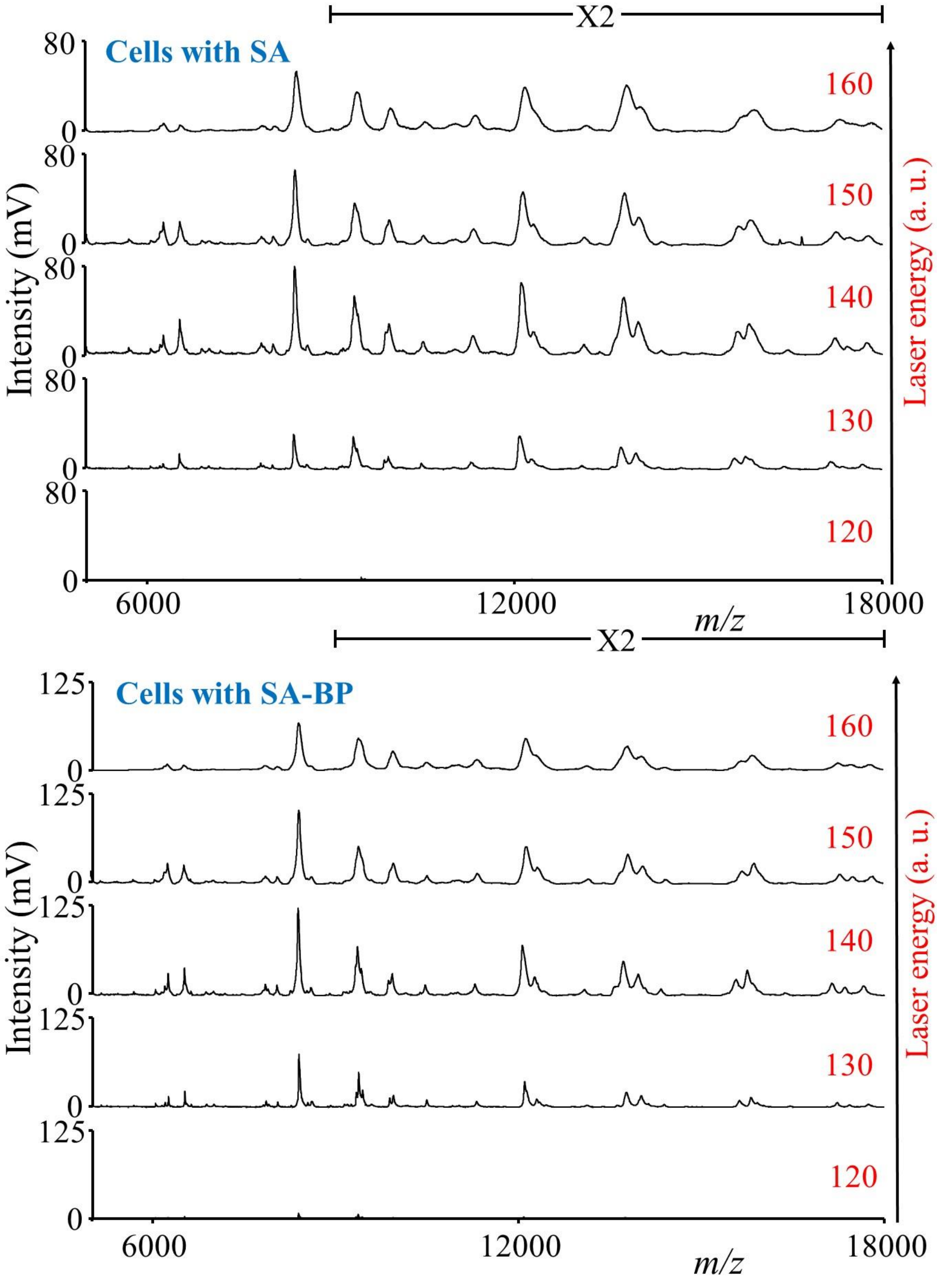

Figure S1. Comparison of laser energy effect for the ionization of biomolecules from intact cells (mouse ovarian surface epithelial cell line, ID8, SCR) in mass spectrometry using SA and SA-BP matrices. Conditions: positive ion mode, *m/z* range 5000-18000, intensity in *m/z* range 9000-18000 magnified 2 times, intensity normalized to 125 mV (cells with SA-BP) and to 80 mV (cells with SA), 100 profiles.

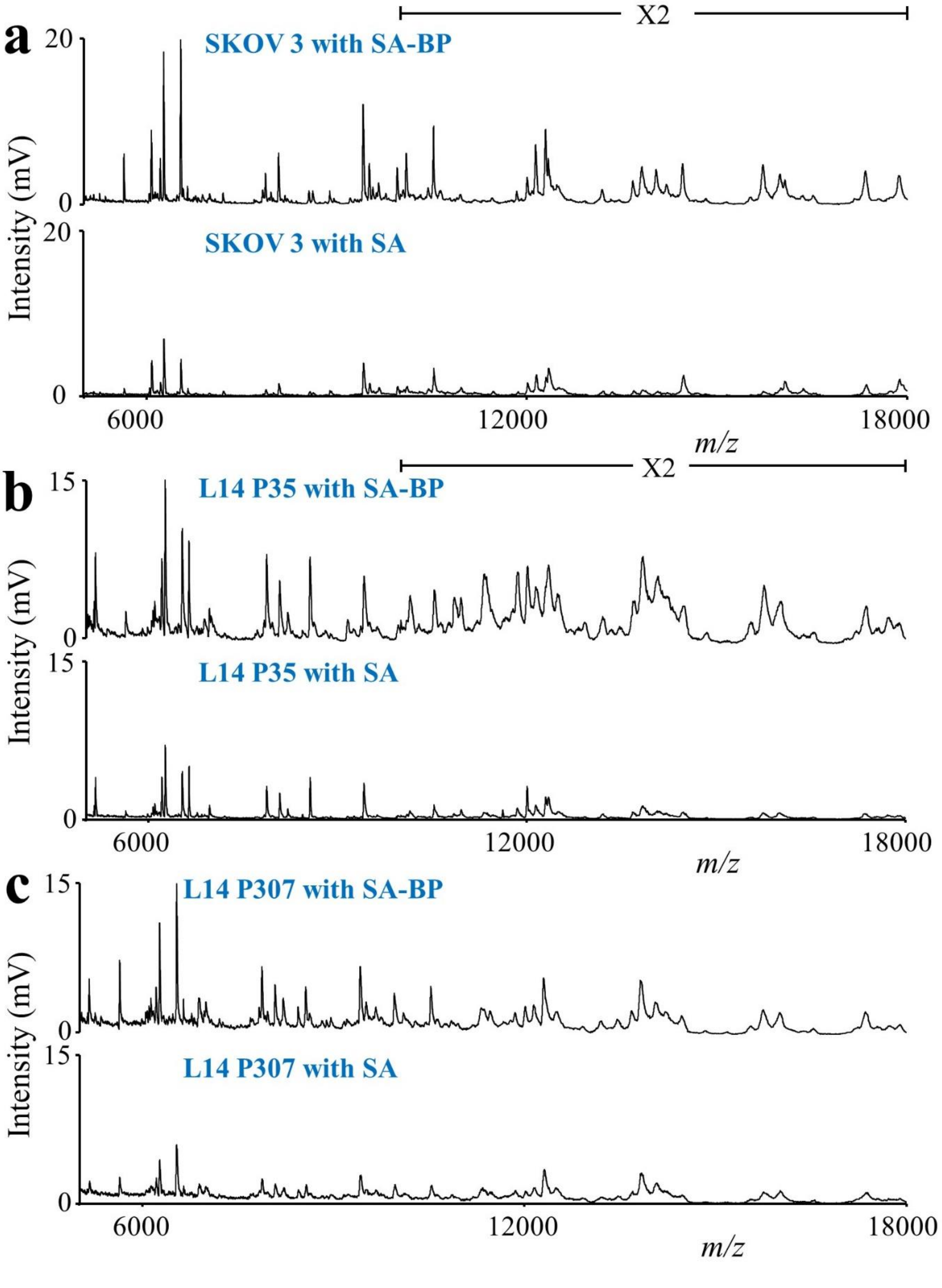

Figure S2. Comparison of mass spectra recorded from intact cells via mass spectrometry using SA and SA-BP matrices. (**a**) human ovarian cancer cell line SKOV 3 (intensity in *m/z* range 10000-18000 magnified 2 times, intensity normalised to 20 mV), (**b**) human embryonic cell line CCTL14 of low passage number P35 (intensity in *m/z* range 10000-18000 magnified 2 times, intensity normalised to 15 mV), and (**c**) human embryonic cell line CCTL14 of high passage number P307 (intensity normalised to 15 mV). Conditions: positive ion mode, laser energy 140 a.u., 100 profiles.

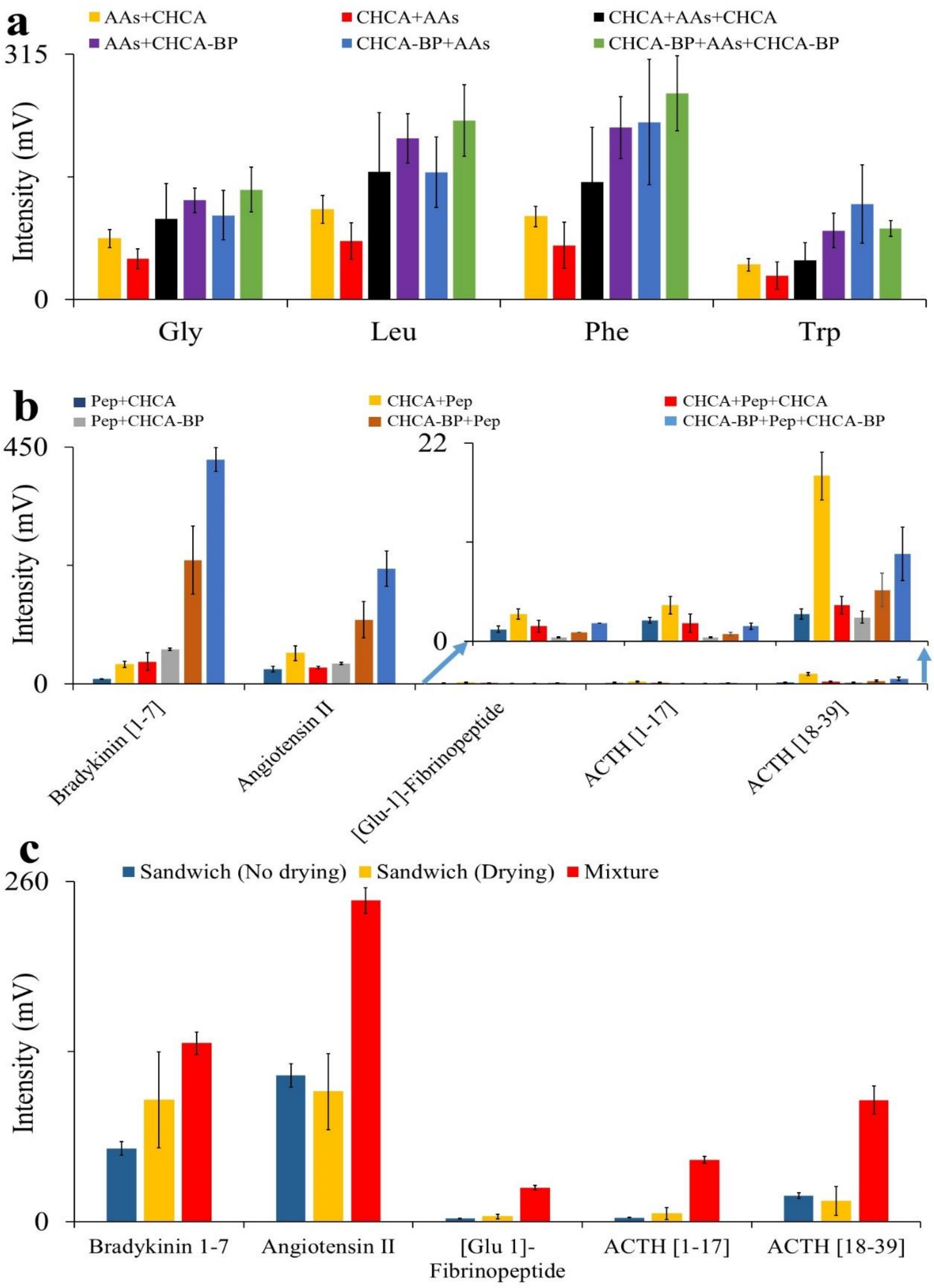

Figure S3. Optimization of sample deposition methods on target. (**a**) amino acids (AAs+CHCA or CHCA-BP, CHCA or CHCA-BP+AAs, CHCA or CHCA-BP+AAs+CHCA or CHCA-BP, n = 3) and (**b**) peptides (Pep+CHCA or CHCA-BP, CHCA or CHCA-BP+Pep, CHCA or CHCA-BP+Pep+CHCA or CHCA-BP, n = 3). Conditions: positive ion mode, laser energy 110 a.u. (for peptides) and 70 a.u. (for amino acids), 100 profiles. (**c**) peptides (Sandwich-No drying after each deposition, Sandwich-Drying after each deposition, and Mixture deposition, n = 5). Conditions: positive ion mode, laser energy 130 a.u., 100 profiles.

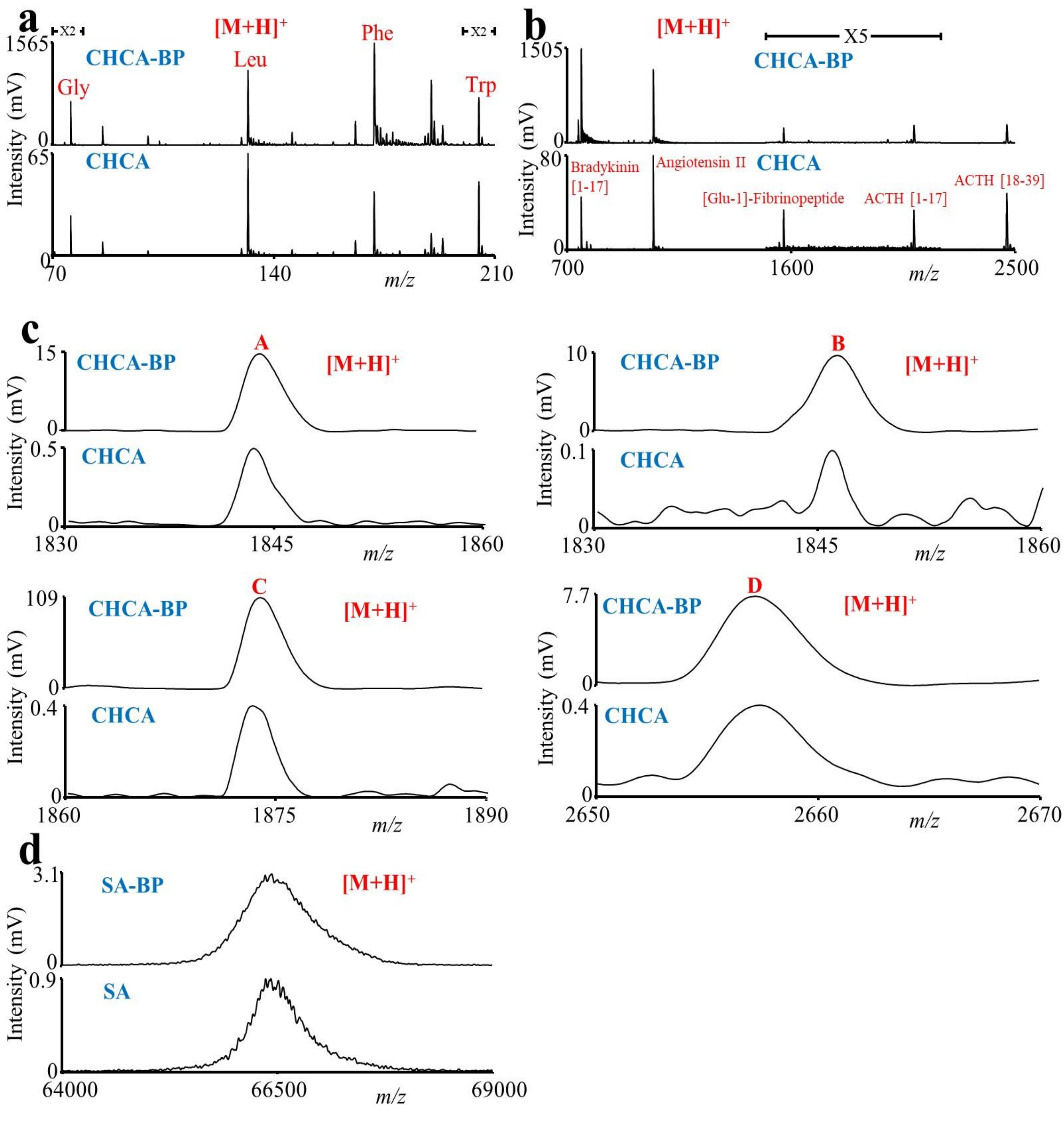

Figure S4. Mass spectra recorded from selected biomolecules using CHCA or SA and BP enriched CHCA or SA matrices. (**a**) amino acids (intensity in *m/z* ranges 70-80 and 200-210 magnified 2 times), (**b**) standard peptides (intensity in *m/z* range 1500-2200 magnified 5 times), (**c**) humanin peptides A, B, C, and D, and (**d**) BSA. Conditions: positive ion mode, laser energy 110 a.u. (for amino acids and peptides) and 180 a.u. (for BSA), 100 profiles.

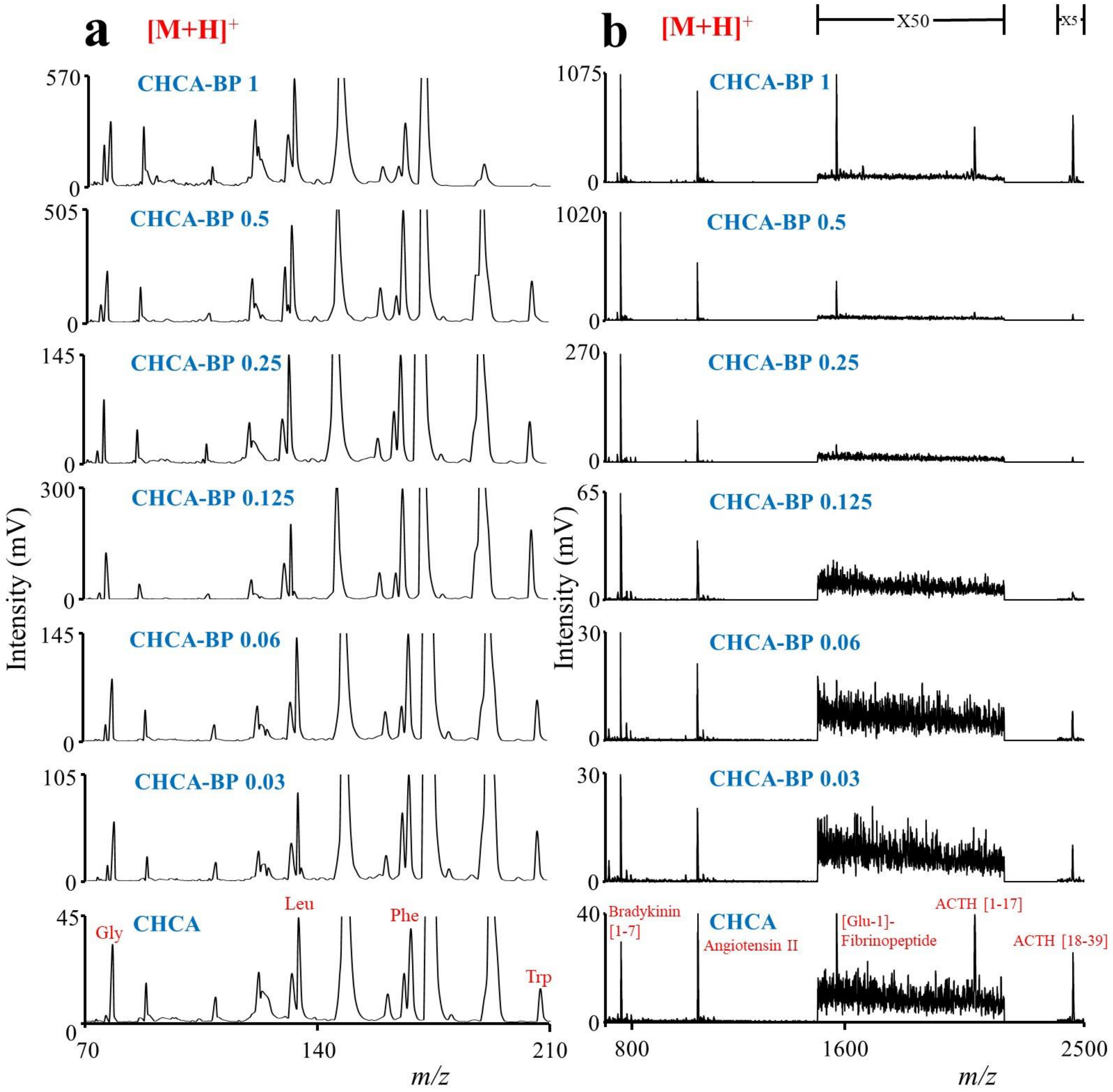

Figure S5. Mass spectra recorded from selected biomolecules using CHCA and BP enriched CHCA matrices during the optimization of BP concentration in CHCA solution for ionization of biomolecules in MALDI TOF MS. (**a**) amino acids (CHCA, CHCA-BP: 0.03, 0.06, 0.125, 0.25, 0.5, and 1 mg BP in 1 mL of CHCA matrix solution), and (**b**) peptides (CHCA, CHCA-BP: 0.03, 0.06, 0.125, 0.25, 0.5, and 1 BP mg in 1 mL of CHCA matrix solution, intensity in *m/z* range 1500-2200 magnified 50 times and intensity in *m/z* range 2400-2500 magnified 5 times). Conditions: positive ion mode, laser energy 110 a.u. (for peptides) and 70 a.u. (for amino acids), 100 profiles.

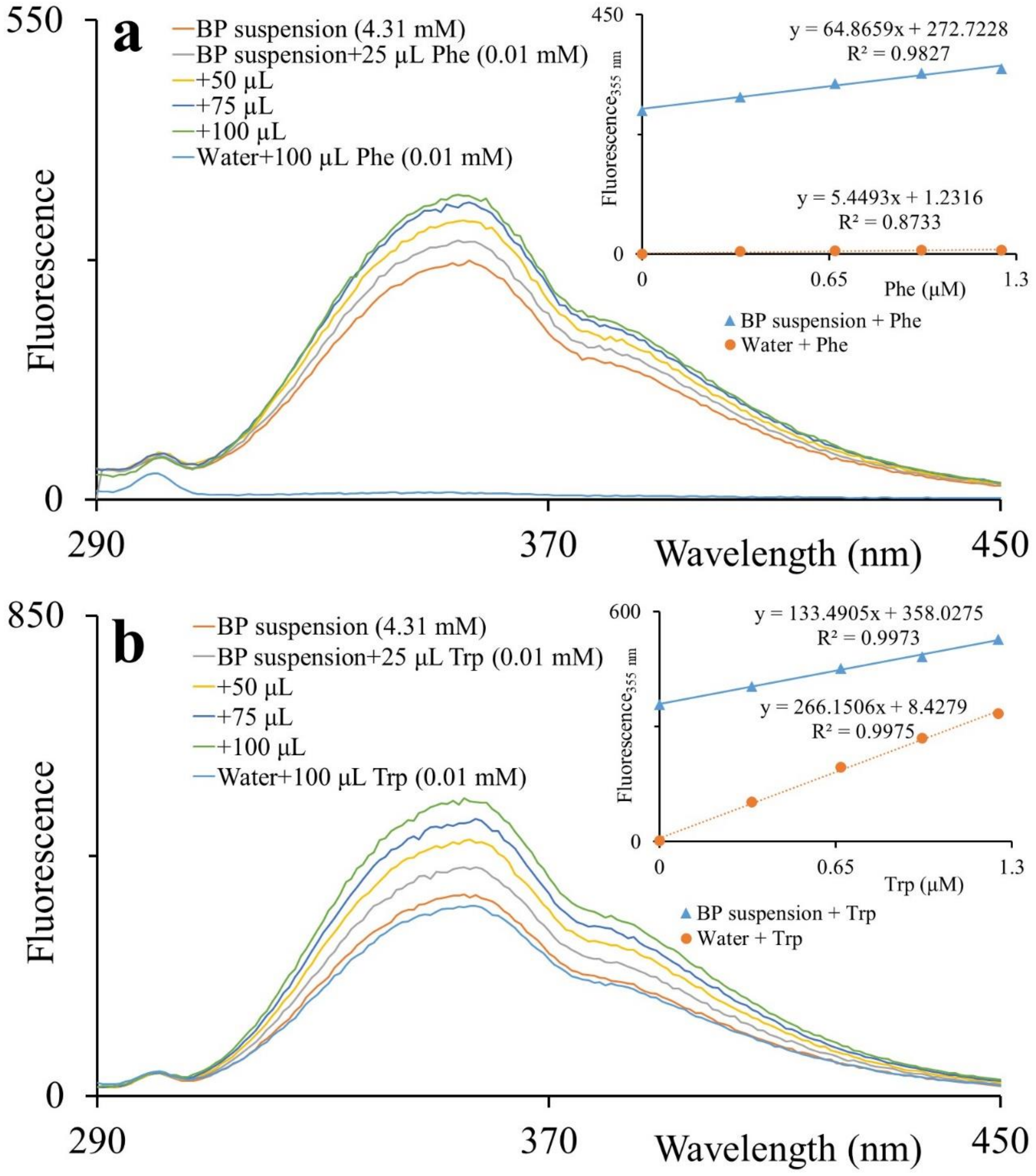

Figure S6. Emission spectra of titrated BP suspension with amino acids. (**a**) phenylalanine and (**b**) tryptophan. The inset shows the plots of emission at 355 nm wavelength against the concentration of BP fine particles in analyte solution (blue line) and in water (orange line).
